# Electron beam processing of freeze-dried and frozen raw pet food to eliminate *Salmonella* and *Listeria monocytogenes*: efficacy and nutritional impact

**DOI:** 10.3389/fvets.2026.1904850

**Published:** 2026-10-29

**Authors:** Neha S. Wavare, Tonali S. Lara-Ramos, Muzaffar Ali, Steven A. Jakeman, Suresh D. Pillai, Chandni Praveen

**Affiliations:** 1 National Center of Electron Beam Research, Texas A&M University, College Station, TX, United States; 2 Department of Food Science and Technology, Texas A&M University, College Station, TX, United States; 3 Carnivore Meat Company LLC, Green Bay, WI, United States

**Keywords:** electron beam, *Listeria monocytogenes*, pet food safety, pet nutrition, raw pet food, *Salmonella* spp.

## Abstract

**Introduction:**

Frozen and freeze-dried raw pet food products are at high risk for the presence of pathogens including *Listeria monocytogenes* and *Salmonella* spp. This study investigated the efficacy of electron beam (eBeam) technology freeze-dried and frozen raw pet food to eliminate pathogen risks and assess whether nutritional composition is impaired.

**Methods:**

Eight different frozen and freeze-dried pet food products representing diverse protein sources and formulations were inoculated with high titer multi serovar cocktails of *L. monocytogenes* and *Salmonella* spp. Products were treated with incremental target eBeam doses of 2.5–25 kGy to determine inactivation kinetics and calculate the D_10_ values in the different matrices. Nutritional impact was assessed at commercially relevant doses of 9.5–12.7 kGy using proximate and macro/ micronutrient profiling.

**Results:**

The D_10_ values for *L. monocytogenes* ranged between 0.65 ± 0.01 kGy and 1.15 ± 0.04 kGy, while those for *Salmonella* spp. ranged between 0.55 ± 0.02 kGy and 1.93 ± 0.10 kGy across the 8 different pet food products. eBeam doses between 5 kGy and 9.5 kGy could effectively achieve >5-log reduction of these key pathogens suggesting that the commercially relevant doses (between 9.5 kGy and 12.7 kGy) were sufficient for substantial microbial inactivation. Proximate composition (moisture, crude protein, crude fat, crude fiber, and ash), mineral content, amino acid profiles, fatty acid profiles, and the majority of vitamins (D3, B1, B_2_) remained statistically unchanged (*q* > 0.05) across all product formats evaluated. Vitamin A and vitamin E were the most sensitive to eBeam treatment in selected products.

**Discussion:**

These findings demonstrate that eBeam processing is an effective non-thermal kill-step for frozen and freeze-dried raw pet food to eliminate *Listeria* spp. and *Salmonella* spp., while preserving the overall nutritional quality. Current commercially employed doses can be expected to achieve >5-log reduction of these pathogens if present in these products.

## 1 Introduction

The pet food industry has seen a paradigm shift in recent decades with pet owners increasingly opting for raw pet food diets as a healthier alternative to traditional kibble and canned foods. Minimal processing and enhanced nutrient retention associated with raw diets are key factors driving this preference (1). Raw pet food, referred to as Raw Meat Based Diets (RMBD) comprises of diets containing uncooked animal tissues which include commercial frozen raw diets, freeze dried products etc. The global raw pet food market has experienced exponential growth, with the freeze-dried dog food market estimated to be USD 2.7 billion in 2024 and projected to reach USD 3.8 billion by 2030 (2). Proponents of this feeding philosophy claim several potential benefits, including improvements in immune function, vitality, skin and coat condition, and the management of chronic digestive, allergic, metabolic disorders and overall health. (3, 4). Raw pet food is also shown to preserve heat sensitive nutrients and amino acids which are substantially reduced with thermal processing methods such as extrusion and retorting (5).

Despite their growing popularity, raw pet food diets raise significant food safety concerns due to their susceptibility to microbial contamination (6). Recent studies have identified microplastic contamination in commercially processed dry pet food also as an emerging safety concern (7, 8), highlighting the need for comprehensive evaluation of all safety risks across different pet food formats. Pets consuming contaminated raw products as well as humans handling these foods are vulnerable to a wide range of infections caused by pathogens (9). Among the pathogens of greatest concern are *Salmonella* spp. and *Listeria monocytogenes*. According to reports from the U.S. Food and Drug Administration (FDA), raw pet food products account for overwhelming majority of FDA pet food recall linked to microbial pathogens. These recalls are predominantly associated with contamination by *Salmonella* and *Listeria* (60). Most recently, multiple raw pet food recalls issued in March and May 2026 involving raw chicken, salmon, beef, turkey, and tripe-based pet food products were attributed to contamination by *L. monocytogenes* and *Salmonella* spp. These recurring incidents underscore the microbiological safety concerns inherent to raw and minimally processed pet food products.

Pets exposed to these pathogens may develop clinical illness; however, they can also act as asymptomatic carriers, shedding the bacteria, and facilitating transmission to humans through close contact (61). *Salmonella* infections can result in serious illness in both animals and humans, and in severe cases may lead to death, especially among vulnerable populations. While many individuals recover without medical intervention, *Salmonella* infection can disseminate from the intestines to blood stream leading to fatal outcomes, among vulnerable populations (61). Although *L. monocytogenes* infections are relatively uncommon in pets, transmission to humans can still occur even in the absence of clinical symptoms in the animal host. In humans, *L. monocytogenes* can cause severe illness, particularly in high-risk populations such as pregnant individuals, young children, the elderly, and immunocompromised people. Collectively these safety concerns highlight the critical role for effective intervention technologies for reducing food borne pathogen risk in raw pet food without compromising the nutrient integrity and characteristics of RMBD.

To date, thermal treatments are the most widely used intervention strategy for reducing microbial risk and extending the shelf life of such pet foods (10, 11). In commercial pet food manufacturing, two primary thermal processing approaches used are retort processing and extrusion. During retort processing the packaged pet food is subjected to high temperatures ranging from 121 °C to 140 °C to achieve sterility and shelf-life stability (12). During extrusion the raw ingredients are exposed to a combination of high temperature (80 °C−200 °C), elevated pressure (34–37 atm) and intense mechanical shear forces within a barrel and screw system (5, 13). While both these approaches effectively reduce microbial hazards, the thermal treatment conditions significantly compromise the nutritional quality and product integrity (5, 14). Proteins in meat products are particularly susceptible to heat-induced structural changes during thermal processing. For example, myoglobin denatures at relatively low temperatures (30 °C−35 °C), while collagen begins to denature at 58 °C−65 °C, leading to alterations in protein structure and color changes that affect the sensory quality of meat products (15, 16). In addition, collagen denaturation can trigger protein hydrolysis and structural modifications that alter texture and may increase toughness under excessive heat treatment (17). The heat-induced changes are fundamentally incompatible with raw pet food products, which depend upon preservation of native protein structures, color and nutrients as their defining product characteristics. Due to these limitations, particularly for raw ingredients such as meat and fish that are rich in heat-sensitive proteins, increasing attention has been directed toward non-thermal sterilization technologies for ensuring microbial safety (18).

Several non-thermal treatment technologies were explored as alternative strategies with High Pressure Processing (HPP) being the most widely adopted technology in the raw pet food industry today (19). HPP achieves pathogen inactivation by subjecting the packaged food products to isostatic pressures of 300–600 MPa for short durations, which leads to disruption of microbial cellular membrane s and protein denaturation (20, 21). Due to its ability to cause minimal damage to organoleptic properties, nutritional quality and functional characteristics, HPP is also widely used in variety of human food products including fruit juices, packaged vegetables, ready to eat (RTE) meat products (22–27). However, HPP treatment is ineffective against bacterial spores, as spore inactivation often requires pressures approaching 600 MPa in combination with temperatures exceeding 100 °C. Additionally, HPP is ineffective in low-moisture or dry food systems because the process typically requires water activity levels greater than 0.8 for efficient pressure transfer and microbial inactivation (28). To overcome this serious limitation of HPP, the basic HPP technology is now offered coupled with high temperature as temperature-assisted HPP (29). Other non-thermal approaches including cold plasma (30), Ultraviolet- C (UV-C) irradiation (31) and chemical antimicrobial washes have been investigated but remain constrained by limited effectiveness in complex food matrix, surface only decontamination capability, variable efficacy and challenges in commercial-scale implementation in raw pet food industry (10).

Ionizing radiation is one of the most effective, scientifically validated and regulatory approved non thermal technology for enhancing microbiological safety while preserving nutritional and sensory qualities of food products (32–34). Use of ionizing radiation for animal and pet food and feed is permitted under US code of federal regulations 21 CFR 579 for pathogen control and shelf-life extension (35). Ionizing radiation inactivates microorganisms through both direct and indirect damage to cells. Energetic electrons cause direct DNA damage by inducing single and double strand breaks, while indirect damage occurs through generation of highly reactive free radicals from water radiolysis (32, 36). Since one has to balance pathogen inactivation with retention of nutritional properties, optimizing the dose is critical to ensure microbial inactivation while preserving nutritional quality. Identifying the optimal dose ranges to achieve these end points is of intellectual property value to the company adopting this technology.

Traditionally, gamma radiation from the radioactive isotope Cobalt-60 has been used as the primary ionizing technology for eliminating pathogens in both human and pet foods. Gamma irradiation technology benefits from several attributes such as over 60 years of commercial experience (with this technology), deep penetration capability allowing treatment options for shipping cases or pallets or large bags that are densely packaged. However, the technology is beset with specific technology challenges such as very poor dose rate which often requires prolonged exposure times to deliver the dose, the 12% loss of specific activity of the isotope resulting in decreasing dose rate as time progresses as well as security challenges of transporting, storing, using and disposal of used cobalt-60 material. From a pet food perspective, the low dose rate has been shown to enhance lipid oxidation and contribute to undesirable off-flavor development in gamma-treated products (37, 38, 62). For all these reasons, gamma irradiation as a technology is slowly losing its appeal in the commercial industry (39).

To overcome these limitations, electricity based ionizing technologies such as eBeam and X-ray are becoming popular as alternatives to gamma technology (39). Equipment termed “accelerators” are used to accelerate electrons from commercial electricity to 99.8% speed of light to create the energetic electrons (36). These machine-based technologies eliminate the need for radioactive materials while offering superior dose rate control, operational flexibility and enhanced processing throughput making them ideally suited for industry scale food safety applications. Regardless of whether the ionizing technology is gamma, eBeam or Xray, ultimately it is the secondary electrons that are responsible for the efficacy of of ionizing technologies. During high energy electron beam (HEEB) processing, energetic electrons are generated from commercial electricity primarily using linear accelerators (LINACs). These LINACs accelerate electrons to near the speed of light at energies ranging from 5–10 MeV (36). Unlike gamma, eBeam technology can be switched on and off as needed and operates at significantly higher dose rate enabling product treatment within s (32). Thus, energetic electrons can achieve the same effects of gamma irradiation within significantly short periods of time yet preserving the raw state, native protein conformation and nutrient quality parameters critical for raw pet food products.

Although the efficacy of eBeam processing is well established in various food commodities, its specific application to RMBD, particularly frozen and freeze-dried pet food matrices, has not been extensively reported. Hence, the information regarding microbial inactivation kinetics and nutritional impacts in these products remains limited in the public domain. Therefore, the objectives of the present study are to: (1) evaluate the effectiveness of eBeam treatment for the inactivation of *Salmonella* spp. and *Listeria monocytogenes* in various freeze-dried and frozen raw meat products, and (2) comprehensively assess the impact of eBeam treatment on nutritional quality of raw pet food products treated at commercially relevant dose ranges.

## 2 Materials and methods

### 2.1 Raw pet food samples

Eight commercially available raw pet food products representing both frozen (F) and freeze dried (FD) formulations were selected for this study. These food products were obtained from Carnivore meat company LLC., Green Bay, WI. The products were selected to represent the diverse range of protein sources (pork, beef, chicken, duck, fish etc.) and product formulations (whole organ, ground formulation, patties, cubes) of commercially available raw pet food (Table 1). Upon sample receipt, frozen products were stored at −18 °C ± 2 °C, and freeze-dried products were stored at ambient room temperatures until use.

**Table 1:** List of raw pet food products evaluated in this study.

Sample ID	Raw pet food product	Protein source	Product format
F-PP	Frozen Pork Patty Formula	Pork	Frozen
FD-BL	Freeze Dried Cubed Beef Liver	Beef (organ)	Freeze Dried
FD-SS	Freeze Dried Salmon Skin	Salmon	Freeze Dried
FD-CH	Freeze Dried Chicken Hearts	Chicken (Organ)	Freeze Dried
FD-WM	Freeze Dried Whole Minnows	Fish (Minnow)	Freeze Dried
FD-DN	Freeze Dried Duck Small Nib Formula	Duck	Freeze Dried
FD-GC	Freeze dried Ground Chicken Formula	Chicken	Freeze Dried - Ground Mass
FD-PP	Freeze Dried Pork Patty Formula	Pork	Freeze Dried

### 2.2 Microbial inactivation kinetics

All sample preparation, packaging, inoculation and microbiological analyses were performed in the Molecular Microbiology laboratory at Texas A&M University (College Station, TX, USA) adhering to the Biosafety Level 2 containment standards in accordance with the TAMU Institutional Biosafety Committee (IBC) regulations.

#### 2.2.1 Bacterial strains and inoculation

A multi-serovar *Salmonella enterica* cocktail (DENT) comprising four serovars: *S*. Dublin (ATCC 39184), *S*. Enteritidis (ATCC 25928), *S*. Newport (ATCC 6962), and *S*. Typhimurium (ATCC 14028) was used in this study. A *Listeria monocytogenes* cocktail consisting of three strains: Li2 (ATCC 19115, Serotype 4b), Lm68 (ATCC BAA-839, Serotype 1/2b), and EGDe (ATCC BAA-679, Serotype 1/2a) was also included. Individual bacterial strains were grown overnight in Tryptic Soy Broth (TSB) (Becton Dickinson, Sparks, MD, USA) at 37 °C. Following incubation, overnight cultures were harvested by centrifugation at 4,000 rpm for 10 min at 4 ° C. The resulting bacterial pellet was washed three times with 0.1% phosphate-buffered saline (PBS) by resuspending the pellet in PBS. After the final wash, the pellet was resuspended in 10 mL of PBS, and the bacterial concentration was determined by serial dilution and standard plate counting on Tryptic Soy Agar (TSA, Becton Dickinson, Sparks, MD, USA) plates. Based on the titer obtained, inoculum volume was adjusted to obtain approximately 10^9^ CFU/ mL. Equal volumes of individual strain suspension were then combined to prepare the *Salmonella* DENT and *Listeria monocytogenes* cocktails, respectively.

Triplicate samples (25 g each) frozen and freeze-dried meat products were separately inoculated with 1 mL of the respective pathogen cocktail to achieve an initial inoculation level of approximately 10^9^ CFU/25 g. Frozen samples were thawed to room temperature before inoculation. The inoculum was applied by pipetting the bacterial cocktail into the samples and injecting as multiple small droplets distributed evenly across the sample to ensure uniform coverage. Inoculated frozen samples were stored at −18 °C ± 2 °C, and freeze-dried products were stored at ambient room temperature for 24 h prior to eBeam treatment to allow the inoculated organisms to adsorb to the meat matrix thereby simulating natural contamination to the maximum extent possible. Uninoculated control samples were also prepared appropriately.

#### 2.2.2 eBeam processing

The eBeam treatment was performed at National Center for Electron Beam Research (NCEBR), Texas A&M University, College Station, TX using 10 MeV 15 kW S band LINAC (L3 Pulse Sciences, Melbourne, FL).

Inoculated samples were packaged using a triple-layer containment system in compliance with TAMU IBC requirement for transportation of biohazardous materials. Samples were double-bagged using Whirl-Pak^®^ bags (Nasco, Fort Atkinson, WI) and subsequently placed in 95 kPa certified transport bags (Therapak^®^, Arlington Heights, IL). The packaged samples were transported to the NCEBR for eBeam processing. Each sample was exposed to incremental target eBeam doses of 0 kGy (Untreated Control), 2.5 kGy, 4 kGy, 7 kGy, 12.5 kGy, and 25 kGy, based on parameters established during dose mapping studies (SI 1). eBeam processing was performed using a dual-beam configuration comprising a top-mounted linear accelerator (LINAC) and a bottom-mounted (pit) LINAC. This dual-beam configuration ensured irradiation from both the top and bottom surfaces of the samples simultaneously, thereby maximizing dose uniformity throughout the sample thickness. Dose mapping was performed to verify a Dose Uniformity Ratio (DUR) close to 1.0, signifying that the minimum and maximum absorbed doses across the package were nearly equivalent. DUR is a key parameter in eBeam processing, as it indicates the uniformity of eBeam dose distribution throughout the entire product and it is critically important in the determination of D_10_ values.

Absorbed doses were measured with calibrated alanine dosimeters (GEX, Palm City, FL) using Bruker E-Scan ESR spectrometer (Bruker, Billerica, MA). Dose Uniformity Ratio (DUR) was assessed to ensure uniform eBeam dose distribution across different sample matrices being treated. All dosimetry is traceable to National Institute of Standards and Technology (NIST) standards.

#### 2.2.3 Microbiological analysis

Following eBeam treatment, treated and control samples were transported back to laboratory and stored for a minimum of 24 h under conditions simulating their typical commercial storage. For microbiological enumeration, samples were aseptically transferred to sterile stomacher bags and homogenized in phosphate-buffered saline (PBS) using a stomacher. The resulting homogenates were serially diluted and plated on selective differential agar media. The *Salmonella enterica* samples were enumerated on Xylose Lysine Deoxycholate (XLD) agar (Hardy Diagnostics Criterion, CA, USA), where characteristic colonies appeared as black centered colonies indicative of hydrogen sulfide production. *L. monocytogenes* was enumerated on Polymyxin Acriflavine Lithium Chloride Ceftazidime Esculin Mannitol (PALCAM) agar (Millipore, Darmstadt, Germany), where characteristic colonies appeared as dark green colonies with black halo from esculin hydrolysis. Uninoculated samples from each product type were also plated to determine the background bioburden.

#### 2.2.4 Microbial inactivation kinetics

To evaluate dose dependent microbial inactivation, surviving bacterial populations were expressed as log CFU/25 g of product. Survival curves were constructed for *L. monocytogenes* and *Salmonella* spp. across all the products by plotting log10 CFU/25 g vs. absorbed dose (kGy). The D_10_ values were calculated as the negative reciprocal of the slope and reported as mean ± SE (*n* = 3). The coefficient of determination (R^2^) was determined to confirm linearity of the inactivation kinetics. For product–pathogen combinations where three or more enumerable plate counts (above the limit of detection) were available across the dose range, D_10_ values were calculated using only these countable data points. However, for combinations exhibiting rapid inactivation, only two enumerable data points were obtained before surviving populations fell below the limit of detection. In these cases, the LOD was included as the terminal data point at the corresponding dose to enable regression analysis. For statistical analysis, paired *t*-tests were applied to compare D_10_ values for *Listeria* and *Salmonella* within each product, while one-way ANOVA was used to assess differences in D_10_ values across products for each pathogen. Statistical significance was set at *p* < 0.05. All linear regression analyses and statistical calculations were performed using GraphPad Prism (version 11.0.1; GraphPad Software Inc., San Diego, CA, USA).

### 2.3 Nutritional quality analysis

To assess the impact of eBeam treatment on the nutritional composition of raw pet food products, comprehensive nutritional analysis was carried out in seven of the eight products listed in Table 1. Freeze-dried salmon skin was excluded from nutrient analysis because its naturally high fat content created challenges during sample preparation. All products were collected from Carnivore Meat Company, and no microbiological inoculation was performed in any of the samples for nutrient analysis. For each product type, six samples were collected, three untreated control and three subjected to eBeam processing at commercially relevant doses of 9.5–12.7 kGy. The eBeam treatment was performed at a commercial processing facility, Next Beam (North Sioux City, SD, USA). Following eBeam treatment, both control and treated samples were shipped to Midwest Laboratories Inc. (Omaha, NE, USA), an accredited third-party analytical laboratory for comprehensive nutrient profiling. A complete panel of macro and micronutrients was evaluated as identified by the Association of American Feed Control Officials (AAFCO). Specific parameters analyzed include Proximate composition, Amino acid profile, Fatty acid composition, Mineral content and Vitamin content etc. All analytical procedures were performed in accordance with standard methods established by AOAC International (Association of Official Analytical Chemists). For each product type, mean values (± standard error) of all nutritional parameters were calculated from triplicate samples for both control and eBeam treated groups. For each product type, statistical comparisons between control and eBeam-treated groups across multiple nutritional parameters were performed using multiple unpaired *t*-tests (one per nutritional parameter) with false discovery rate (FDR) correction using the two-stage linear step-up procedure of Benjamini, Krieger, and Yekutieli to control the FDR at 5% (40). This approach was employed to prevent inflation of the Type I error rate associated with performing multiple simultaneous comparisons across numerous nutritional parameters (41). A discovery (statistically significant difference) was identified when the FDR adjusted *q* value was less than 0.05. All statistical analyses were conducted using GraphPad Prism (version 11.0.1; GraphPad Software Inc., San Diego, CA, USA).

## 3 Results

### 3.1 Microbial inactivation kinetics

eBeam inactivation studies demonstrated a clear dose-dependent reduction of *L. monocytogenes* and *Salmonella* spp. across all frozen and freeze-dried meat matrices evaluated. Log-linear survival curves were generated by plotting microbial populations as a function of increasing eBeam dose. Table 2 is a compilation of the D_10_ values of the two pathogens in the 8 different commercially available products. Across all product types, a decrease in pathogen counts were observed with increasing eBeam dose, confirming the efficacy of eBeam treatment as a non-thermal intervention for microbial inactivation. The inactivation kinetics varied slightly depending on the product matrix, likely due to differences in composition, water activity, and physical structure. The eBeam inactivation kinetics for each product type tested are presented in Figures 1A–H.

**Table 2:** Decimal Dose Reduction (D_10_ value) and Estimated eBeam doses required to achieve a 5-log reduction of *Listeria monocytogenes* and *Salmonella* DENT cocktail in raw pet products.

Raw pet food product	Decimal dose reduction (D_10_) value (kGy)	
	*Listeria monocytogenes* cocktail	*Salmonella DENT* cocktail
Frozen Pork Patty Formula	0.83 ± 0.07^A^	0.55 ± 0.02^A^†
Freeze Dried Cubed Beef Liver	0.81 ± 0.05^A^	1.18 ± 0.16^B^
Freeze Dried Salmon Skin	0.82 ± 0.09^A^†	0.59 ± 0.17^A^†
Freeze Dried Chicken Hearts	0.81 ± 0.01^A^†	0.86 ± 0.01^A^†
Freeze Dried Whole Minnows	0.97 ± 0.14^A^	1.12 ± 0.05^B^
Freeze Dried Duck Small Nib Formula	1.15 ± 0.04^B^	1.11 ± 0.04^B^
Freeze Dried Ground Chicken Formula	0.65 ± 0.01^A^	1.93 ± 0.10^C^
Freeze Dried Pork Patty Formula	0.69 ± 0.00^A^	1.13 ± 0.05^B^

D_10_ values are represented as Mean± Standard Error. Within each column, values sharing the same superscript letter are not significantly different (*p* > 0.05). Different superscript letters (A, B, C) within the same column indicate statistically significant differences in D10 values across product matrices for that pathogen (ANOVA, *p* < 0.05). Superscript comparisons are column-wise only and do not indicate comparisons between pathogens. † D_10_ value calculated using the LOD as the terminal data point as a conservative estimate.

**Figure 1:**
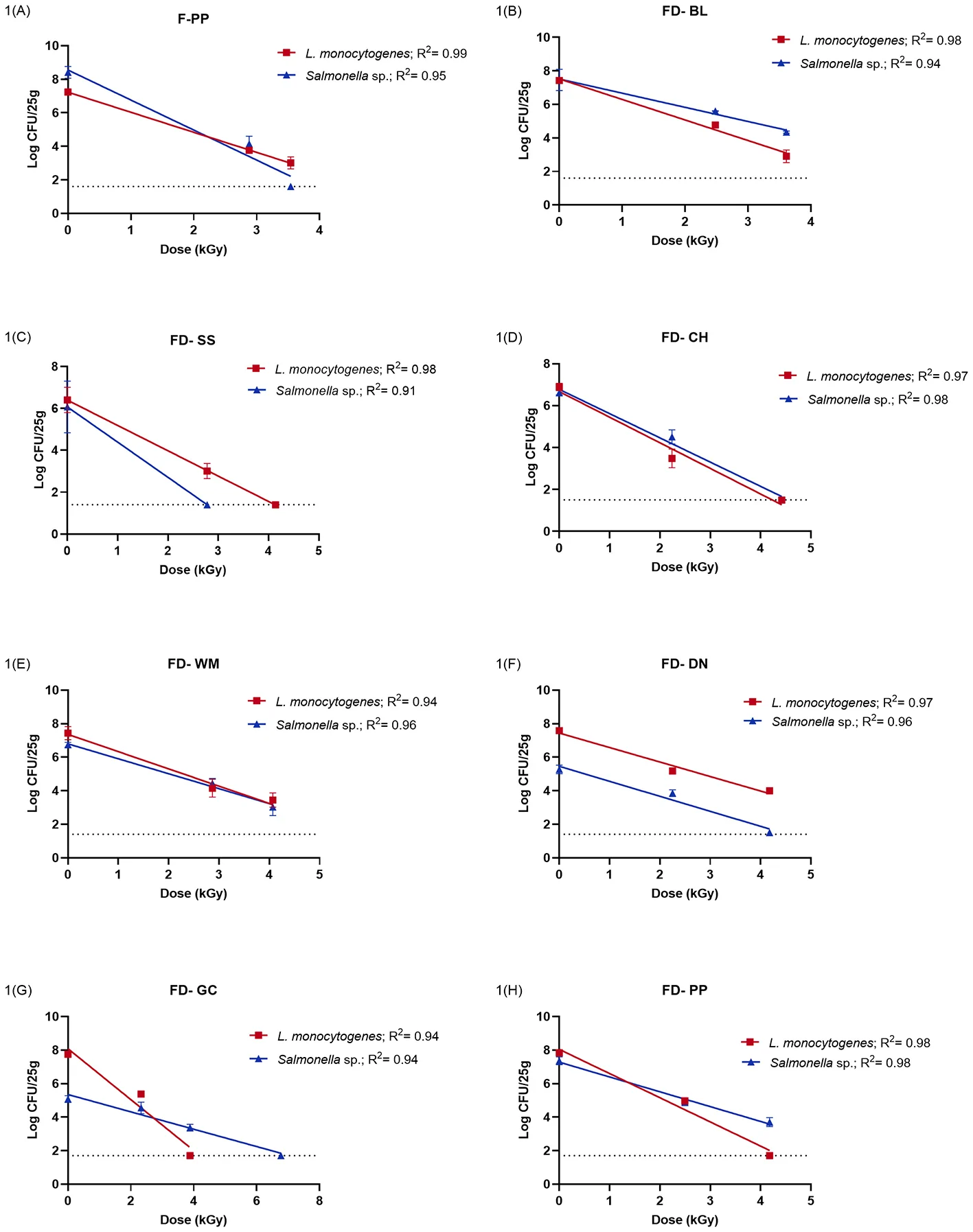
Inactivation kinetics of *L. monocytogenes* and *Salmonella* DENT cocktail in various frozen and [[Inline Image]]freeze-dried raw pet food matrices following incremental eBeam doses. (A-H) represent different pet food products: **(A)** frozen pork patty formula, **(B)** freeze dried cubed beef liver, **(C)** freeze dried Salmon skin, **(D)** freeze dried chicken hearts, **(E)** freeze dried whole minnows, **(F)** freeze dried duck small nib formula, **(G)** freeze dried ground chicken formula, and **(H)** freeze dried pork patty formula. Pathogen survivor counts (Log CFU/25 g) are plotted as a function of absorbed eBeam doses (kGy). Linear regression curves are presented for inactivation kinetics along with corresponding R^2^ values to illustrate the goodness-of-fit. Detection limits are indicated by a dotted horizontal line. Data represent mean ± standard error (*n*=3).

The D_10_ value, defined as the eBeam dose (kGy) required to achieve a 1-log (90%) reduction in microbial population, was used to characterize pathogen inactivation kinetics across different raw pet food matrices. D_10_ value serves as a quantitative measure of microbial susceptibility to eBeam treatment and enables direct comparison across different products matrices with varying pathogen titers. Lower D_10_ value indicates greater sensitivity to eBeam treatment and more efficient pathogen inactivation. In this study, D_10_ values were determined for both *L. monocytogenes* cocktail and the *Salmonella* DENT cocktail in all 8 raw pet food products (Table 2).

For the *L. monocytogenes* cocktail, D_10_ values ranged from 0.65 ± 0.01 kGy to 1.15 ± 0.04 kGy across the products tested, indicating narrow range of variation among matrices. In comparison, the *Salmonella* DENT cocktail D_10_ values were more variable and ranged from 0.55 ± 0.02 kGy to 1.93 ± 0.10 kGy suggesting strong matrix dependent response. No statistically significant differences were observed between *Salmonella* spp. and *L. monocytogenes* D_10_ values within the same product. However, one-way ANOVA demonstrated that D_10_ values of *Salmonella* spp. varied significantly across different product matrices (*p* < 0.05), with freeze dried products such as FD-BL, FD-GC and FD-PP exhibiting notably higher resistance to eBeam. In contrast, D_10_ values of *L. monocytogenes* remained relatively consistent across different products (Table 2).

Comparison of the frozen and freeze-dried pork patty formulations highlighted the influence of product moisture on microbial susceptibility to eBeam treatment. *Salmonella* was more sensitive in frozen pork patty (0.55± 0.02 kGy) than freeze-dried pork patty (1.13± 0.05 kGy). Among all the products tested, freeze dried duck nibs showed relative higher D_10_ value for both *L. monocytogenes* and *Salmonella* with a D_10_ of 1.15± 0.04 and 1.11± 0.04 respectively. The microbiological studies of uninoculated products revealed that none of the products tested had *Salmonella* spp. and *L. monocytogenes* in the background microbial bioburden.

### 3.2 Nutritional analysis

Nutritional analysis was conducted for seven food matrices to assess the effect of commercially relevant eBeam doses (9.5–12.7 kGy) on nutrient composition compared to untreated control. Proximate composition of nutrients was used as a primary indicator of the overall nutritional quality of the product. This assessment includes moisture, crude protein, crude fat, crude fiber and ash and helps to determine whether the eBeam processing has altered overall nutrient profile. As shown in Table 3, eBeam processing resulted in minimal changes in proximate composition across all tested matrices. Following FDR corrected multiple comparisons, no statistically significant differences (q <0.05) were observed between control and eBeam-treated groups for any proximate composition parameter across all seven product formats. Crude protein, fat and ash content were preserved in eBeam treated samples compared to control, indicating retention of macronutrients and mineral fractions. Crude protein ranged from 48.30 ± 0.66% (F-PP, eBeam) to 76.10 ± 0.68% (FD-CH, Control) and fat content varied substantially across product types, ranging from 10.03 ± 0.38% (FD-BL, Control) to 46.33 ± 2.29% (F-PP, eBeam), reflecting differences in formulation and protein source, but remained statistically comparable between control and eBeam-treated groups across all products (*q* > 0.05). Although variation was observed in crude fiber, the magnitude of these differences was limited and could be in part due to the analytical variability of the method used.

**Table 3:** Proximate composition (%) of freeze-dried (FD-BL, FD-WM, FD-CH, FD-GC, FD-DN, FD-PP) and frozen (F-PP) raw pet food products before (control) and after eBeam treatment at commercially relevant doses (9.5–12.7 kGy).

Nutritional components (Dry weight basis)	FD- BL		FD- WM		FD- CH		FD- GC	
	Control	eBeam	Control	eBeam	Control	eBeam	Control	eBeam
Moisture (%)	9.25 ± 0.41	8.51 ± 0.14	6.57 ± 0.17	6.11 ± 0.13	4.6 ± 0.08	4.06 ± 0.09	5.29 ± 0.31	4.60 ± 0.09
Crude Protein (%)	70.50 ± 0.52	69.70 ± 0.70	68.47 ± 0.15	68.60 ± 0.67	76.10 ± 0.68	73.43 ± 2.03	59.97 ± 0.32	60.27 ± 0.07
Fat (%)	10.03 ± 0.38	11.47 ± 0.75	13.70 ± 0.95	13.47 ± 0.64	19.90 ± 2.00	20.80 ± 0.23	32.80 ± 1.35	35.83 ± 0.55
Crude fiber (%)	0.15 ± 0.15	0.39 ± 0.22	0.00 ± 0.00	1.35 ± 0.37	0.42 ± 0.21	1.5 ± 0.36	0.99 ± 0.15	0.35 ± 0.18
Ash (%)	4.34 ± 0.16	5.46 ± 0.18	13.77 ± 0.15	13.80 ± 0.2	3.35 ± 0.13	4.21 ± 0.02	4.64 ± 0.13	4.61 ± 0.05
**Nutritional components (Dry weight basis)**	**FD-DN**		**FD-PP**		**F-PP**			
	**Control**	**eBeam**	**Control**	**eBeam**	**Control**	**eBeam**			
Moisture (%)	5.1 ± 0.04	5.16 ± 0.04	5.42 ± 0.37	4.90 ± 0.16	70.6 ± 0.93	71.10 ± 0.20			
Crude Protein (%)	57.77 ± 0.39	58.67 ± 0.29	48.93 ± 0.72	49.97 ± 0.42	50.70 ± 3.05	48.30 ± 0.66			
Fat (%)	26.73 ± 0.66	28.37 ± 0.38	44.73 ± 1.33	43.37 ± 0.47	34.37 ± 0.43	46.33 ± 2.29			
Crude fiber (%)	0.20 ± 0.10	1.33 ± 0.18	0.20 ± 0.20	1.49 ± 0.16	1.22 ± 0.05	2.66 ± 0.64			
Ash (%)	12.27 ± 0.79	12.73 ± 0.09	3.37 ± 0.03	4.00 ± 0.13	4.05 ± 0.95	3.48 ± 0.14			

FD-BL, freeze-dried Cubed Beef liver; FD-WM, freeze dried Whole Minnows; FD-CH, freeze dried Chicken hearts; FD-GC, freeze dried Ground Chicken formula; FD-PP, freeze-dried Pork patty formula; F-PP, frozen pork patty formula. Values are expressed as mean ± Standard Error (n=3). Statistical significance between control and eBeam-treated groups was determined using multiple unpaired *t*-tests with FDR correction. No statistically significant differences (*q* > 0.05) were observed for any proximate composition parameter.

Additionally, detailed nutrient profiling was performed according to AAFCO- relevant parameters, including Amino acids, fatty acids, minerals and vitamins (SI 2). Selected key nutrient profiles are shown in Table 4. Overall, the results showed that eBeam processing preserved major nutritional components across all the tested matrices. The Amino acids, fatty acids and minerals remained largely comparable to the untreated control across all matrices evaluated. Taurine and lysine, selected as key nutritional markers for companion animals, showed no statistically significant differences between control and eBeam-treated groups in any product format (*q* > 0.05). Taurine content ranged from 0.12 ± 0.00% (FD-GC, Control) to 1.06 ± 0.01% (FD-CH, eBeam), while lysine ranged from 3.52 ± 0.04% (F-PP, eBeam) to 5.99 ± 0.14% (FD-CH, eBeam). The omega-3 polyunsaturated fatty acids DHA and EPA also remained statistically unchanged across all products (q > 0.05). Although numerical reductions in DHA (~17.4%) and EPA (~14.3%) were observed in the fish-based product FD-WM, these did not reach statistical significance following FDR correction.

**Table 4:** Major nutrient components of freeze-dried (FD-BL, FD-WM, FD-CH, FD-GC, FD-DN, FD-PP) and frozen (F-PP) raw pet food products before (control) and after eBeam treatment at commercially relevant doses (9.5–12.7 kGy).

Nutritional components (Dry weight basis)	FD- BL		FD- WM		FD- CH		FD- GC	
	Control	eBeam	Control	eBeam	Control	eBeam	Control	eBeam
Minerals								
Calcium (%)	0.02 ± 0.00	0.03 ± 0.00	3.43 ± 0.12	3.44 ± 0.17	0.92 ± 0.00	0.89 ± 0.05	0.88 ± 0.06	0.69 ± 0.03
Phosphorus (%)	1.26 ± 0.01	1.24 ± 0.01	2.52 ± 0.05	2.41 ± 0.08	0.05 ± 0.00	0.15 ± 0.07	1.00 ± 0.04	0.88 ± 0.02
Vitamins								
Vitamin A (IU/kg)	3483.33 ± 66.92	2913.33 ± 378.17	1960.00 ± 98.49	1150.00 ± 92.92	1166.67 ± 24.04	929.33 ± 14.11	30733.33 ± 240.37	24066.67 ± 520.68
Vitamin B1 (mg/kg)	10.20 ± 0.78	9.30 ± 0.40	3.99 ± 0.17	3.98 ± 0.15	5.70 ± 0.03	5.48 ± 0.15	3.66 ± 0.13	3.76 ± 0.38
Vitamin B2 (mg/kg)	5.51 ± 0.23	5.72 ± 0.29	0.47 ± 0.01	0.51 ± 0.02	2.57 ± 0.03	2.57 ± 0.06	0.59 ± 0.01	0.58 ± 0.01
Vitamin D3 (IU/kg)	23.93 ± 6.32	36.73 ± 3.27	13566.67 ± 218.58	12533.33 ± 600.93	114.83 ± 12.61	145.00 ± 15.57	491.33 ± 9.61	507.00 ± 13.75
Vitamin E (IU/kg)	0.00 ± 0.00	0.00 ± 0.00	78.27 ± 1.23	26.33 ± 0.63^*^	27.47 ± 15.88	0.00 ± 0.00	498.00 ± 23.71	418.00 ± 14.42
Amino acids								
Taurine (%)	0.13 ± 0.00	0.14 ± 0.01	0.85 ± 0.01	0.92 ± 0.02	1.01 ± 0.01	1.06 ± 0.01	0.12 ± 0.00	0.13 ± 0.00
Lysine (%)	4.29 ± 0.26	4.74 ± 0.06	5.21 ± 0.13	5.08 ± 0.18	5.91 ± 0.26	5.99 ± 0.14	5.31 ± 0.06	5.20 ± 0.09
Fatty acids								
DHA g/100g	0.02 ± 0.01	0.03 ± 0.00	1.21 ± 0.07	1.00 ± 0.07	0.02 ± 0.00	0.02 ± 0.00	0.10 ± 0.00	0.10 ± 0.00
EPA g/100g	0.02 ± 0.00	0.03 ± 0.00	0.70 ± 0.05	0.60 ± 0.03	0.01 ± 0.00	0.01 ± 0.01	0.13 ± 0.00	0.14 ± 0.00
Nutritional components (Dry weight basis)	**FD- DN**		**FD- PP**		**F-PP**			
	Control	eBeam	Control	eBeam	Control	eBeam		
Minerals								
Calcium (%)	3.34 ± 0.35	3.22 ± 0.17	0.40 ± 0.10	0.41 ± 0.20	0.28 ± 0.14	0.35 ± 0.04		
Phosphorus (%)	1.96 ± 0.15	1.84 ± 0.08	0.82 ± 0.05	0.80 ± 0.08	0.70 ± 0.05	0.79 ± 0.01		
Vitamins								
Vitamin A(IU/kg)	167666.67 ± 1333.33	124666.67 ± 881.92^*^	510333.33 ± 14193.11	405000.00 ± 11060.44	424666.67 ± 42915.94	304000.00 ± 38070.11^**^		
Vitamin B1 (mg/kg)	5.24 ± 0.12	5.24 ± 0.02	6.02 ± 0.45	6.38 ± 0.08	8.61 ± 0.37	7.49 ± 0.36		
Vitamin B2 (mg/kg)	1.81 ± 0.01	1.5 0± 0.05	2.95 ± 0.08	2.61 ± 0.13	21.77 ± 0.44	25.40 ± 1.56		
Vitamin D3 (IU/kg)	3843.33 ± 14.53	3376.67 ± 39.30	637.33 ± 11.17	632.67 ± 19.53	656.67 ± 30.75	630.00 ± 21.17		
Vitamin E (IU/**k**g)	22.73 ± 8.80	37.43 ± 1.01	80.13 ± 4.43	68.57 ± 1.31	274.67 ± 30.61	122.67 ± 4.18		
Amino acids								
Taurine (%)	0.37 ± 0.01	0.35 ± 0.00	0.13 ± 0.00	0.14 ± 0.01	0.20 ± 0.01	0.24 ± 0.00		
Lysine (%)	3.87 ± 0.27	4.01 ± 0.06	3.69 ± 0.08	3.60 ± 0.06	3.78 ± 0.10	3.52 ± 0.04		
Fatty acids								
DHA (g/100g)	0.07 ± 0.00	0.08 ± 0.00	0.04 ± 0.00	0.04 ± 0.00	0.00 ± 0.00	0.00 ± 0.00		
EPA (g/100)	0.09 ± 0.00	0.09 ± 0.00	0.04 ± 0.00	0.05 ± 0.00	0.01 ± 0.00	0.01 ± 0.00		

FD-BL, freeze-dried Cubed Beef liver; FD-WM, freeze dried Whole Minnows; FD-CH, freeze dried Chicken hearts; FD-GC, freeze dried Ground Chicken formula; FD-PP, freeze-dried Pork patty formula; F-PP, frozen pork patty formula. Values are expressed as mean ± Standard Error (n=3). Statistical significance between control and eBeam-treated groups was determined using multiple unpaired t-tests with false discovery rate (FDR) correction using the two-stage linear step-up procedure of Benjamini, Krieger, and Yekutieli. Significance was determined based on q-values: ^*^ q <0.05; ^**^ q <0.01.

Among the nutrients assessed, vitamins were found to be the most sensitive nutrient class to eBeam treatment. Vitamin A and Vitamin E were the most consistently affected nutrients. Following FDR correction, statistically significant reductions in Vitamin A were observed in two of the tested products, freeze dried Duck nibs (~25.6%) and frozen Pork patty formula (~28.4%). Vitamin E showed a statistically significant reduction in freeze dried Whole Minnows (~66.4%). Vitamin D3 remained largely stable across most products, with only modest reductions in freeze dried whole Minnows (~7.6%) and freeze dried duck nibs (~12.1%), that did not reach statistical significance. Water soluble B vitamins (thiamine and riboflavin) were well preserved across all product formats following eBeam treatment, with no statistically significant changes detected. Overall, these findings indicate that eBeam treatment maintained the nutritional quality of the raw pet food products, with only limited reductions observed in selected vitamin components.

## 4 Discussion

This study provides experimental evidence suggesting that eBeam processing is an effective non thermal intervention for microbial inactivation without impairing the nutritional attributes in the raw pet food industry. Current knowledge on non-thermal intervention technologies for microbial inactivation is primarily limited to HPP. HPP is currently the most widely adopted technology for pathogen inactivation in raw and frozen pet foods. Previous studies have shown that HPP applied alone, or in combination with lactic acid treatment, or coupled with extended freezing, can effectively reduce *L. monocytogenes, Salmonella* spp. and STEC *E. coli* in raw pet food (21, 42, 43). HPP is found to be effective for raw and frozen pet foods, where high moisture content facilitates pressure transmission and pathogen inactivation (44). However, HPP faces substantial limitations when applied to low-moisture formats such as freeze-dried products. The freeze drying process removes water from the product, resulting in poor pressure transmission and suboptimal microbial inactivation (21). Consistent with this limitation, Perez-Baltar et al. (45) reported limited inactivation of *L. monocytogenes* in dry-cured ham using HPP. Lee et al. (42) demonstrated that even in high-moisture raw pet food matrices, the efficacy of HPP varied considerably among pathogens, with *L. monocytogenes* requiring substantially longer pressure exposure times for microbial reduction, highlighting the pathogen-specific variability in HPP efficacy.

This study conclusively demonstrates that eBeam processing is equally effective in both frozen and freeze-dried raw pet food matrices. D_10_ values for *L. monocytogenes* and *Salmonella* DENT cocktails ranged from 0.65 kGy to 1.15 kGy and 0.55 kGy to 1.93 kGy, respectively. A slight increase in D_10_ value for *Salmonella* was observed in the freeze-dried pork patty (1.13 ± 0.05 kGy) compared to the frozen pork patty (0.55 ± 0.02 kGy). However, this trend was not observed for *Listeria* (0.69 ± 0.00 kGy for freeze-dried vs. 0.83 ± 0.07 kGy for frozen), and the differences were not statistically significant. The microbial D_10_ values observed are in line with the established efficacy of eBeam processing in meat and other protein rich food systems (46). Several studies have consistently demonstrated dose dependent, rapid inactivation of pathogens using eBeam as well as gamma technology. Thayer et al. (47) and Chung et al. (48) documented 2.8-5 log CFU/g reduction of *Salmonella typhimurium* in poultry or beef at doses as low as 1.5-3 kGy using eBeam or gamma treatment. Park et al. (49) showed that eBeam and gamma technology were both efficient in a variety of meat matrices with no adverse effect in quality and sensory characteristics, emphasizing the flexibility of these technologies across product types. Studies by Brahmakshatriya et al. (50) and Jeong et al. (51) found that eBeam treatment effectively inactivated avian influenza virus in poultry and duck meat without quality loss, underscoring its applicability across diverse pathogens and species.

Table 5 presents the minimum eBeam dose required to achieve a validated 5-log reduction in *L. monocytogenes* and *Salmonella* spp. in different commercially relevant raw pet food matrices The minimum eBeam dose based on respective D_10_ values were 3.25-5.75 kGy for *Listeria* and 2.75-9.65 kGy for *Salmonella*, which are well within operational ranges reported by other researchers in different meat products (34, 47, 48, 52, 53). Notably, these dose ranges are lower than the commercial relevant dose used in this study (9.5-12.7 kGy), highlighting that the selected eBeam dosing conditions are adequate to achieve 5-log pathogen reduction threshold for a broader range of raw pet product matrices. This underscores the robustness of eBeam processing in microbial inactivation yet also ensures compliance with the US FDA zero- tolerance policy for *Salmonella* in pet food and treats (54).

**Table 5:** Estimated eBeam doses required to achieve a 5-log reduction of *Listeria monocytogenes* and *Salmonella* DENT cocktail in raw pet products.

Raw pet food product	eBeam dose required for 5 log reduction (kGy)	
	*Listeria monocytogenes* cocktail	*Salmonella DENT* cocktail
Frozen pork patty formula	4.15	2.75
Freeze dried cubed beef liver	4.05	5.9
Freeze dried salmon skin	4.10	2.95
Freeze dried chicken hearts	4.05	4.30
Freeze dried whole minnows	4.85	5.6
Freeze dried duck small nib formula	5.75	7.9
Freeze dried ground chicken formula	3.25	9.65
Freeze dried pork patty formula	3.45	5.65

Nutritional preservation is a critical factor when evaluating processing technologies for pet food products. Lack of statistically significant changes in the proximate composition (crude protein, crude fat, crude fiber, moisture, and ash) following eBeam treatment at 9.5-12.7 kGy confirms preservation of the nutrient profile of raw pet food products. The stability of crude protein and amino acid profiles suggests minimal protein cleavage or structural alteration at the applied eBeam dose. Preservation of all essential and non-essential amino acids is critical as protein quality and amino acid bioavailability is crucial for pet nutrition (55). Similarly, crude fat and other fatty acid profiles of eBeam processed samples also confirmed that at the targeted dose range measurable loss in lipid profile is absent. However, it is important to note that the use of higher eBeam doses can potentially induce oxidative damage, which may negatively influence both protein integrity and lipid stability (53). As observed in many previous studies, vitamins constituted the most sensitive nutrient class to eBeam treatment, especially vitamin A and E (53, 56–58). Studies have shown that while vitamins B2, B6, B12, folic acid exhibited more stability, vitamin A, B1, C and E were more prone to radiolytic cleavage and oxidation during eBeam or gamma treatment (58). Importantly, while the statistically significant reductions in Vitamins A and E are noteworthy, their practical nutritional significance must be interpreted in the context of complete diet formulation. Commercial raw pet food products are typically formulated to exceed minimum AAFCO nutrient requirements, providing a nutritional buffer that can accommodate moderate processing related losses. The extent of loss remains minor relative to the overall nutrient profile and can generally be compensated for in product formulation.

The nutritional impact assessment in this study was conducted at commercially relevant doses of 9.5-12.7 kGy to reflect real world processing conditions. However, the D10 values obtained indicate that a 5-log reduction of both *Salmonella* and *L. monocytogenes* could be achieved at substantially lower doses (approximately 3.25-9.65 kGy depending on the matrix). Given that radiation induced vitamin degradation is generally dose-dependent (56, 63), it is reasonable to anticipate that vitamin losses, particularly for Vitamins A and E would be proportionally lower at these reduced effective doses. Future dose optimization studies evaluating the nutritional impact at the minimum effective dose for each specific product format are warranted.

While this study demonstrates effectiveness of eBeam processing, we acknowledge certain limitations and opportunities for future research. First, the study focused primarily on bacterial pathogens such as *L. monocytogenes* and *Salmonella* spp., so the results cannot be directly extrapolated to viral pathogens, including emerging threats like High Pathogenic Avian Influenza (59). Although viral inactivation was not directly assessed in this study, past studies from our lab (50) and recent studies (51) show that eBeam treatment can effectively reduce avian influenza in meat products. Brahmakshatriya et al. (50) reported a D_10_ value of 2.6 kGy for avian influenza virus (H5N3) in ground turkey meat, while Jeong et al. (51) found a D_10_ value of approximately 2.34 kGy for H5N3 in shredded duck breast meat. Based on these published studies, it is plausible that the dose range used in the present study (9.5–12.7 kGy) may contribute to viral load reduction. However, this remains to be experimentally validated. Future studies should directly evaluate the efficacy of eBeam processing for viral inactivation in raw pet food matrices. Second, the nutritional impact was assessed only at commercially relevant doses (9.5–12.7 kGy), dose optimization studies evaluating the nutritional impact at the minimum effective dose for each product format would further strengthen the practical application of the eBeam technology. The long-term stability of nutrient and shelf-life studies post eBeam processing were not evaluated in the present study and warrant future investigation. Third, while macronutrient composition, amino acid profiles, fatty acid profiles, and mineral content remained stable following eBeam treatment, lipid oxidation markers (e.g., TBARS, peroxide value), instrumental color measurements, and sensory/palatability evaluations were beyond the scope of this initial investigation. These quality parameters represent important next steps, and follow-up studies incorporating TBARS analysis, color evaluation, water activity assessment, volatile profiling are currently being planned to provide a comprehensive quality assessment of eBeam treated raw pet food products.

## 5 Conclusion

Our findings confirmed that eBeam processing is effective at eliminating risks from microbial pathogens in raw pet food products. The ability of eBeam technology to achieve targeted pathogen reduction by adjusting the dose is significant from a commercial usability standpoint. Furthermore, eBeam technology was able to achieve the reduction irrespective of the type of raw freeze-dried pet food. D_10_ values did not differ significantly between frozen and freeze-dried products, supporting the conclusion that eBeam is broadly effective regardless of product type or moisture status. Despite the substantial microbial reductions achieved, eBeam treatment preserved the overwhelming majority of the nutritional profile at commercially relevant eBeam doses of 9.5–12.7 kGy. This, combined with the fact that eBeam can be applied post-packaging, positions it as an ideal terminal decontamination method for the pet food industry. Furthermore, eBeam can be integrated into production lines for post-packaging treatment, either in-house or through third-party providers, supporting both flexibility and scalability for pet food manufacturers of various sizes. These benefits directly support commercial-scale adoption and enhance product safety to meet the growing demand for minimally processed raw pet food products.

## Data Availability

The original contributions presented in the study are included in the article/Supplementary material, further inquiries can be directed to the corresponding author.
