# Supplementary material for: Electron beam processing of freeze-dried and frozen raw pet food to eliminate Salmonella and Listeria monocytogenes: efficacy and nutritional impact
Source: Front Vet Sci. 2026 Oct 29;13:1904850. doi: 10.3389/fvets.2026.1904850 (PMC13756593; doi:10.3389/fvets.2026.1904850)
Supplement: Supplementary file 1 [file Data_Sheet_1.pdf]

Supplementary Information

S1- Dose mapping and dose uniformity ratio

Sample	Minimum Absorbed Dose (D_{min})	Maximum Absorbed Dose (D_{max})	Dose Uniformity Ratio (DUR)
Frozen Pork Patty	0.89	0.94	**1.05**
Freeze dried Beef cubes	0.79	0.88	**1.11**
Freeze dried Salmon Skin	0.84	0.95	**1.13**
Freeze dried Chicken heart	0.86	0.93	**1.08**
Freeze dried Minnows	0.87	0.93	**1.07**
Freeze dried Duck nibs	0.89	0.94	**1.06**
Freeze dried Ground chicken mix-in	0.85	0.9	**1.06**
Freeze dried Pork patty	0.88	0.93	**1.06**

Sample Type	Target Dose	Speed (fpm)	Calculated Minimum Dose (kGy)	Calculated Maximum Dose (kGy)	Dose Uniformity Ratio (DUR)
**Freeze Dried Beef Cubes**	2.5 kGy	60.00	2.35	2.61	**1.05**
	4 kGy	36.50	3.41	3.80	
	7 kGy	20.85	5.99	6.67	
	12.5 kGy	11.65	10.72	11.95	
	25 kGy	5.80	21.93	24.43	
**Frozen Pork Patty**	2.5 kGy	60.00	2.80	2.96	**1.11**
	4 kGy	41.15	3.44	3.63	
	7 kGy	23.50	6.24	6.59	
	12.5 kGy	13.15	10.53	11.12	
	25 kGy	6.55	21.32	22.51	
**Salmon skin**	2.5 kGy	60.0	2.61	2.95	**1.13**
	4 kGy	35.2	3.88	4.39	
	7 kGy	20.1	6.55	7.41	
	12.5 kGy	11.2	12.70	14.36	
	25 kGy	5.60	24.25	27.43	
**Chicken Hearts**	2.5 kGy	60.0	2.15	2.33	**1.08**
	4 kGy	35.2	4.24	4.59	
	7 kGy	20.1	7.13	7.71	
	12.5 kGy	11.2	12.27	13.27	
	25 kGy	5.60	26.09	28.21	
**Minnows**	2.5 kGy	60.0	2.77	2.97	**1.07**
	4 kGy	35.2	3.93	4.20	
	7 kGy	20.1	6.99	7.47	
	12.5 kGy	11.2	12.28	13.12	
	25 kGy	5.60	24.78	26.49	
**Duck Nibs**	2.5 kGy	60.00	2.18	2.31	**1.06**
	4 kGy	37.30	4.06	4.29	
	7 kGy	21.30	6.77	7.15	
	12.5 kGy	11.90	12.02	12.70	

	25 kGy	5.95	24.70	26.08	
**Chicken Mix**	2.5 kGy	60.00	2.26	2.39	**1.06**
	4 kGy	37.30	3.77	3.99	
	7 kGy	21.30	6.57	6.96	
	12.5 kGy	11.90	12.49	13.23	
	25 kGy	5.95	24.59	26.03	
**Pork Patty (freeze-dried)**	2.5 kGy	60.00	2.43	2.56	**1.06**
	4 kGy	37.30	4.06	4.29	
	7 kGy	21.30	7.11	7.52	
	12.5 kGy	11.90	12.85	13.58	
	25 kGy	5.95	24.12	25.49	

S2- Complete Nutrient profile of eBeam proceesed and control raw pet food products

Minerals

Nutritional components (Dry weight basis)	FD-BL		FD-WM		FD-CH		FD-GC	
	Untreated	Treated	Untreated	Treated	Untreated	Treated	Untreated	Treated
Sulphur (total) AR%	0.71± 0.01	0.73± 0.00	0.77± 0.00	0.78± 0.01	0.96± 0.00	0.93± 0.02	0.55± 0.01	0.55± 0.01
Sulphur (total) DW%	0.78± 0.01	0.80± 0.00	0.82± 0.01	0.83± 0.01	1.01± 0.00	0.97± 0.02	0.58± 0.01	0.58± 0.01
Magnesium (total) AR%	0.06± 0.00	0.24± 0.18	0.13± 0.00	0.14± 0.00	0.08± 0.00	0.08± 0.00	0.08± 0.00	0.08± 0.00
Magnesium (total) DW%	0.05± 0.02	0.07± 0.00	0.14± 0.00	0.15± 0.00	0.08± 0.00	0.08± 0.00	0.08± 0.00	0.08± 0.00
Calcium (total) AR%	0.02± 0.00	0.03± 0.00	3.35± 0.16	3.23± 0.15	0.05± 0.00	0.14± 0.07	0.83± 0.06	0.66± 0.03
Calcium (total) DW%	0.02± 0.00	0.03± 0.00	3.43± 0.12	3.44± 0.17	0.05± 0.00	0.15± 0.07	0.88± 0.06	0.69± 0.03
Sodium (total) AR%	0.20± 0.00	0.22± 0.01	0.39± 0.00	0.42± 0.00	0.27± 0.00	0.29± 0.01	0.22± 0.00	0.23± 0.00
Sodium (total) DW%	0.22± 0.00	0.24± 0.01	0.42± 0.00	0.45± 0.00	0.28± 0.00	0.30± 0.01	0.23± 0.00	0.24± 0.00
Iron (total) AR%	177.67± 4.48	179.67± 11.61	116.23± 20.91	103.00± 1.73	151.33± 3.71	151.33± 9.40	119.67± 5.78	122.67± 2.85
Iron (total) DW%	195.67± 5.24	196.33± 12.25	124.67± 22.70	109.67± 1.76	159.00± 4.04	157.67± 9.74	129.67± 4.98	128.67± 2.85
Manganese (total) AR ppm	9.80± 0.26	10.23± 0.12	17.30± 0.64	17.63± 0.76	11.57± 8.72	3.23± 0.49	6.63± 0.98	8.37± 2.07
Manganese (total) DW ppm	10.80± 0.26	11.17± 0.15	18.50± 0.67	18.77± 0.83	3.00± 0.12	3.37± 0.52	7.00± 1.04	8.77± 2.18
Copper (total) AR ppm	248.33± 6.49	251.33± 4.67	2.93± 0.07	3.93± 0.24	14.03± 0.15	14.80± 0.40	8.03± 0.73	9.47± 0.33
Copper (total) DW ppm	271.33± 4.67	274.67± 5.55	3.13± 0.07	4.17± 0.27	14.70± 0.15	15.43± 0.44	8.50± 0.76	9.93± 0.35
Chloride AR %	0.36± 0.00	0.37± 0.01	0.46± 0.01	0.48± 0.01	0.31± 0.01	0.33± 0.02	0.19± 0.01	0.19± 0.01
Chloride DW %	0.39± 0.01	0.40± 0.01	0.50± 0.01	0.51± 0.01	0.32± 0.01	0.34± 0.02	0.20± 0.01	0.20± 0.01
Iodine (total) AR ppm	0.22± 0.02	0.27± 0.01	2.07± 0.22	2.10± 0.20	0.00± 0.00	0.00± 0.00	1.27± 0.03	1.24± 0.01
Iodine (total) DW ppm	0.24± 0.02	0.30± 0.01	2.21± 0.23	2.24± 0.21	0.00± 0.00	0.00± 0.00	1.34± 0.03	1.30± 0.01
Selenium (total) AR ppm	1.76± 0.13	2.00± 0.05	2.25± 0.04	2.05± 0.03	1.40± 0.02	1.30± 0.01	0.78± 0.02	0.79± 0.02
Selenium (total) DW ppm	1.94± 0.14	2.19± 0.06	2.41± 0.03	2.18± 0.03	1.47± 0.03	1.36± 0.01	0.82± 0.02	0.83± 0.02

Nutritional components (Dry weight basis)	FD-DN		FD-PP		F-PP	
	Control	eBeam	Control	eBeam	Control	eBeam
Sulphur (total) AR%	0.60± 0.01	0.61± 0.01	0.49± 0.00	0.50± 0.02	0.14± 0.01	0.15± 0.00
Sulphur (total) DW%	0.63± 0.01	0.64± 0.01	0.52± 0.00	0.53± 0.02	0.48± 0.01	0.53± 0.01
Magnesium (total) AR%	0.10± 0.00	0.07± 0.03	0.06± 0.00	0.06± 0.00	0.01± 0.00	0.02± 0.00
Magnesium (total) DW%	0.10± 0.00	0.10± 0.00	0.06± 0.00	0.06± 0.00	0.05± 0.01	0.07± 0.00
Calcium (total) AR%	3.17± 0.33	3.06± 0.16	0.38± 0.10	0.39± 0.19	0.08± 0.04	0.10± 0.01
Calcium (total) DW%	3.34± 0.35	3.22± 0.17	0.40± 0.10	0.41± 0.20	0.28± 0.14	0.35± 0.04
Iron (total) AR%	120.00± 3.06	121.00± 1.53	284.00± 3.06	284.67± 7.67	75.33± 1.62	90.93± 0.62
Iron (total) DW%	126.33± 3.18	127.33± 1.76	300.33± 4.06	299.33± 7.84	257.00± 5.69	314.33± 2.73
Manganese (total) AR ppm	8.70± 1.80	6.53± 0.09	2.33± 0.09	2.40± 0.10	0.00± 0.00	0.00± 0.00
Manganese (total) DW ppm	9.17± 1.92	6.90± 0.12	2.43± 0.09	2.50± 0.10	0.00± 0.00	0.00± 0.00
Copper (total) AR ppm	23.20± 0.51	23.13± 0.24	31.63± 0.35	32.10± 0.90	8.73± 0.09	10.40± 0.12
Copper (total) DW ppm	24.47± 0.55	24.40± 0.26	33.47± 0.41	33.77± 0.95	29.80± 0.66	35.97± 0.48
Chloride AR %	0.96± 0.02	0.86± 0.03	0.26± 0.01	0.28± 0.00	0.10± 0.00	0.11± 0.00
Chloride DW %	1.01± 0.02	0.90± 0.04	0.27± 0.01	0.30± 0.00	0.35± 0.02	0.37± 0.02
Iodine (total) AR ppm	0.50± 0.00	0.49± 0.01	0.38± 0.10	0.14± 0.01	0.05± 0.05	0.05± 0.05
Iodine (total) DW ppm	0.53± 0.00	0.52± 0.01	0.40± 0.11	0.15± 0.01	1.22± 1.22	0.16± 0.16
Selenium (total) AR ppm	0.97± 0.02	0.96± 0.02	1.15± 0.02	1.13± 0.03	0.30± 0.01	0.31± 0.00
Selenium (total) DW ppm	1.02± 0.02	1.01± 0.02	1.22± 0.03	1.18± 0.03	1.04± 0.06	1.06± 0.02

Amino acids

Nutritional components (Dry weight basis)	FD-BL		FD-WM		FD-CH		FD-GC	
	Untreated	Treated	Untreated	Treated	Untreated	Treated	Untreated	Treated
Alanine AR%	3.24± 0.17	3.23± 0.06	3.78± 0.08	3.45± 0.04	4.15± 0.08	3.81± 0.09	3.54± 0.04	3.51± 0.06
Alanine DW%	3.24± 0.33	3.52± 0.06	3.87± 0.12	3.68± 0.04	4.35± 0.09	3.97± 0.09	3.68± 0.04	3.68± 0.07
Aspartic acid AR%	5.01± 0.30	5.26± 0.11	5.82± 0.23	5.36± 0.03	6.64± 0.08	6.12± 0.04	5.40± 0.05	5.31± 0.05
Aspartic acid DW%	5.52± 0.31	5.59± 0.10	6.23± 0.23	5.71± 0.04	6.96± 0.07	6.38± 0.04	5.70± 0.07	5.57± 0.06
Glutamic acid AR%	6.75± 0.34	7.11± 0.17	7.95± 0.29	7.50± 0.11	10.73± 0.38	9.68± 0.05	2.90± 2.57	5.40± 2.66
Glutamic acid DW%	7.44± 0.34	7.77± 0.17	8.50± 0.29	7.98± 0.11	11.27± 0.42	10.10± 0.06	8.70± 0.13	8.63± 0.20
Glycine AR%	3.36± 0.14	3.04± 0.03	4.08± 0.22	3.70± 0.14	3.73± 0.09	3.42± 0.10	3.34± 0.04	3.22± 0.04
Glycine DW%	3.70± 0.14	3.32± 0.03	4.36± 0.23	3.94± 0.15	3.91± 0.09	3.56± 0.11	3.53± 0.04	3.38± 0.05
Histidine AR%	1.38± 0.03	1.54± 0.05	1.40± 0.06	1.47± 0.03	1.59± 0.06	1.60± 0.02	1.83± 0.02	1.73± 0.01
Histidine DW%	1.53± 0.03	1.68± 0.06	1.50± 0.07	1.50± 0.00	1.66± 0.06	1.67± 0.02	1.93± 0.02	1.81± 0.01
Isoleucine AR%	1.84± 0.11	2.31± 0.11	1.93± 0.11	2.24± 0.04	2.33± 0.12	2.95± 0.17	2.59± 0.02	2.27± 0.10
Isoleucine DW%	2.03± 0.12	2.57± 0.14	2.06± 0.11	2.24± 0.04	2.44± 0.13	3.08± 0.18	2.73± 0.01	2.38± 0.11
Leucine AR%	4.58± 0.15	5.10± 0.03	4.05± 0.15	4.01± 0.10	5.18± 0.11	5.53± 0.10	4.28± 0.05	4.17± 0.05
Leucine DW%	5.05± 0.14	5.57± 0.05	4.33± 0.16	4.27± 0.10	5.43± 0.11	5.76± 0.10	4.18± 0.31	4.30± 0.07
Methionine AR%	1.35± 0.01	1.41± 0.01	1.50± 0.03	1.50± 0.02	1.86± 0.02	1.79± 0.02	1.38± 0.01	1.41± 0.01
Methionine DW%	1.49± 0.01	1.55± 0.01	1.61± 0.03	1.60± 0.02	1.95± 0.02	1.86± 0.02	1.46± 0.02	1.48± 0.01
Phenylalanine AR%	2.61± 0.09	3.08± 0.13	2.28± 0.10	2.42± 0.05	2.53± 0.06	2.88± 0.06	2.03± 0.02	1.34± 0.63
Phenylalanine DW%	2.87± 0.10	3.40± 0.17	2.43± 0.10	2.57± 0.06	2.65± 0.06	3.01± 0.06	2.15± 0.02	1.41± 0.66
Proline AR%	2.47± 0.28	1.94± 0.25	2.44± 0.07	2.04± 0.13	3.02± 0.14	2.31± 0.23	2.54± 0.03	1.69± 0.80
Proline DW%	2.72± 0.30	2.12± 0.27	2.61± 0.07	2.17± 0.15	3.17± 0.15	2.41± 0.23	2.68± 0.02	1.76± 0.84
Serine AR%	2.51± 0.13	2.25± 0.10	2.64± 0.08	2.23± 0.11	3.13± 0.03	2.35± 0.19	2.09± 0.05	2.23± 0.05

Serine DW%	2.77± 0.13	2.46± 0.10	2.82± 0.08	2.37± 0.11	3.28± 0.03	2.44± 0.19	2.21± 0.06	2.34± 0.05
Threonine AR%	2.19± 0.12	2.33± 0.01	2.41± 0.10	2.25± 0.12	3.01± 0.07	2.71± 0.10	2.31± 0.04	1.58± 0.75
Threonine DW%	2.42± 0.13	2.54± 0.01	2.58± 0.10	2.40± 0.13	3.15± 0.07	2.83± 0.11	2.44± 0.05	1.65± 0.79
Tyrosine AR%	1.94± 0.03	1.79± 0.30	1.74± 0.06	1.74± 0.04	2.37± 0.07	2.40± 0.01	1.93± 0.01	1.27± 0.60
Tyrosine DW%	2.13± 0.02	2.32± 0.06	1.86± 0.06	1.85± 0.05	2.48± 0.07	2.51± 0.01	2.04± 0.01	1.33± 0.63
Valine AR%	2.66± 0.29	3.34± 0.03	2.22± 0.10	2.54± 0.04	2.75± 0.11	3.38± 0.11	2.79± 0.01	1.89± 0.61
Valine DW%	2.59± 0.03	3.65± 0.04	2.37± 0.10	2.71± 0.05	2.88± 0.12	3.53± 0.12	2.95± 0.01	11.34± 8.83

Nutritional components (Dry weight basis)	FD-DN		FD-PP		F-PP	
	Control	eBeam	Control	eBeam	Control	eBeam
Alanine AR%	3.52± 0.13	3.46± 0.04	2.96± 0.06	2.90± 0.05	0.85± 0.02	0.76± 0.01
Alanine DW%	3.70± 0.14	3.65± 0.04	3.13± 0.07	3.05± 0.05	2.90± 0.07	2.62± 0.06
Aspartic acid AR%	4.46± 0.29	4.35± 0.04	4.25± 0.08	3.95± 0.04	1.22± 0.04	1.13± 0.01
Aspartic acid DW%	4.70± 0.31	4.58± 0.04	4.50± 0.10	4.16± 0.04	4.18± 0.13	3.92± 0.07
Glutamic acid AR%	7.26± 0.48	7.43± 0.03	6.57± 0.10	6.46± 0.09	1.86± 0.05	1.77± 0.02
Glutamic acid DW%	7.66± 0.50	7.83± 0.03	6.94± 0.13	6.80± 0.08	6.35± 0.05	6.11± 0.09
Glycine AR%	4.74± 0.20	4.52± 0.14	3.24± 0.09	3.14± 0.08	0.88± 0.04	0.81± 0.02
Glycine DW%	4.99± 0.21	4.77± 0.14	3.43± 0.11	3.30± 0.08	2.99± 0.08	2.82± 0.09
Histidine AR%	1.11± 0.09	1.18± 0.01	1.23± 0.03	1.21± 0.02	0.37± 0.01	0.35± 0.00
Histidine DW%	1.17± 0.09	1.24± 0.01	1.30± 0.03	1.28± 0.02	1.27± 0.00	1.20± 0.02
Isoleucine AR%	1.88± 0.25	1.86± 0.34	1.82± 0.07	1.99± 0.05	0.50± 0.03	0.45± 0.01
Isoleucine DW%	1.98± 0.27	2.26± 0.02	1.92± 0.08	2.09± 0.05	1.72± 0.08	1.55± 0.05
Leucine AR%	3.55± 0.28	3.83± 0.01	3.72± 0.06	3.73± 0.04	1.06± 0.01	0.99± 0.02

Leucine DW%	3.74± 0.30	4.03± 0.01	3.93± 0.07	3.93± 0.03	3.63± 0.07	3.43± 0.06
Phenylalanine AR%	1.90± 0.14	1.90± 0.01	1.97± 0.04	1.87± 0.03	0.58± 0.02	0.54± 0.01
Phenylalanine DW%	2.00± 0.15	2.00± 0.01	2.08± 0.05	1.97± 0.03	1.98± 0.02	1.87± 0.04
Proline AR%	3.02± 0.14	3.19± 0.07	2.20± 0.05	2.36± 0.03	0.70± 0.02	0.62± 0.02
Proline DW%	3.19± 0.15	3.37± 0.07	2.33± 0.05	2.49± 0.03	2.39± 0.04	2.16± 0.06
Serine AR%	2.00± 0.08	1.65± 0.16	1.75± 0.08	1.39± 0.11	0.56± 0.03	0.55± 0.00
Serine DW%	2.10± 0.08	1.73± 0.17	1.85± 0.08	1.46± 0.11	1.92± 0.07	1.89± 0.02
Threonine AR%	2.04± 0.16	1.92± 0.09	1.85± 0.02	1.67± 0.08	0.57± 0.02	0.55± 0.01
Threonine DW%	2.14± 0.17	2.02± 0.09	1.95± 0.01	1.84± 0.12	1.95± 0.04	1.90± 0.02
Tyrosine AR%	1.54± 0.15	1.64± 0.01	1.53± 0.03	1.49± 0.02	0.43± 0.01	0.41± 0.01
Tyrosine DW%	1.62± 0.15	1.73± 0.01	1.61± 0.04	1.56± 0.03	1.47± 0.02	1.42± 0.02
Valine AR%	2.21± 0.27	2.48± 0.03	2.35± 0.07	2.41± 0.03	0.64± 0.03	0.56± 0.02
Valine DW%	2.32± 0.29	2.62± 0.03	2.49± 0.08	2.54± 0.03	2.17± 0.08	1.95± 0.07

Fatty acids

Nutritional components (Dry weight basis)	FD-BL		FD-WM		FD-CH		FD-GC	
	Untreated	Treated	Untreated	Treated	Untreated	Treated	Untreated	Treated
Butyric (C4:0)AR g/100g	0.00± 0.00	0.00± 0.00	0.00± 0.00	0.00± 0.00	0.00± 0.00	0.00± 0.00	0.00± 0.00	0.00± 0.00
Caproic (C6:0)AR g/100g	0.00± 0.00	0.01± 0.00	0.00± 0.00	0.00± 0.00	0.00± 0.00	0.00± 0.00	0.00± 0.00	0.00± 0.00
Caprylic (C8:0)AR g/100g	0.00± 0.00	0.00± 0.00	0.00± 0.00	0.00± 0.00	0.00± 0.00	0.00± 0.00	0.00± 0.00	0.00± 0.00
Capric (C10:0)AR g/100g	0.00± 0.00	0.00± 0.00	0.00± 0.00	0.00± 0.00	0.00± 0.00	0.00± 0.00	0.00± 0.00	0.00± 0.00
Lauric (C12:0)AR g/100g	0.00± 0.00	0.00± 0.00	0.01± 0.00	0.01± 0.00	0.00± 0.00	0.00± 0.00	0.01± 0.00	0.01± 0.00
Tridecanoic(C13:0)AR g/100g	0.00± 0.00	0.00± 0.00	0.00± 0.00	0.00± 0.00	0.00± 0.00	0.00± 0.00	0.00± 0.00	0.00± 0.00
Myristic(C14:0)AR g/100g	0.13± 0.05	0.05± 0.01	0.24± 0.02	0.24± 0.01	0.07± 0.01	0.08± 0.00	0.31± 0.01	0.26± 0.09
Myristoledic(C14:1 Trans)AR g/100g	0.00± 0.00	0.00± 0.00	0.00± 0.00	0.00± 0.00	0.00± 0.00	0.00± 0.00	0.00± 0.00	0.00± 0.00
Myristoleic(C14:1 cis)AR g/100g	0.02± 0.00	0.02± 0.00	0.00± 0.00	0.00± 0.00	0.02± 0.00	0.03± 0.00	0.06± 0.00	0.06± 0.00
Pentadecanoic(C15:0)AR g/100g	0.03± 0.00	0.03± 0.00	0.09± 0.01	0.09± 0.00	0.01± 0.00	0.01± 0.00	0.03± 0.00	0.05± 0.02
Palmitic(C16:0)AR g/100g	1.69± 0.31	1.40± 0.12	2.73± 0.19	2.70± 0.11	4.03± 0.41	4.26± 0.04	7.96± 0.36	8.78± 0.15
Palmiteladic(C16:1 Trans)AR g/100g	0.00± 0.00	0.00± 0.00	0.00± 0.00	0.00± 0.00	0.00± 0.00	0.00± 0.00	0.00± 0.00	0.00± 0.00
Palmitoleic(C16:1 Cis)AR g/100g	0.08± 0.02	0.06± 0.01	0.87± 0.03	0.85± 0.01	0.89± 0.02	0.05± 0.00	0.19± 0.00	0.27± 0.01
Heptadecanoic(C17:0)AR g/100g	0.10± 0.01	0.12± 0.01	0.25± 0.02	0.23± 0.01	0.02± 0.00	0.03± 0.00	0.22± 0.17	0.06± 0.00
10-Heptadecanoic(C17:1)AR g/100g	0.00± 0.00	0.00± 0.00	0.00± 0.00	0.00± 0.00	0.00± 0.00	0.00± 0.00	0.00± 0.00	0.00± 0.00
Stearic(C18:0)AR g/100g	3.10± 0.16	4.17± 0.24	0.56± 0.03	0.59± 0.04	2.55± 0.28	2.53± 0.02	2.15± 0.09	2.37± 0.04
Eliadic(C18:1 Trans)AR g/100g	0.17± 0.01	0.21± 0.03	0.05± 0.00	0.05± 0.00	0.04± 0.00	0.05± 0.00	0.07± 0.00	0.08± 0.00
Oleic(C18:1 Cis)AR g/100g	1.84± 0.32	1.44± 0.14	3.42± 0.24	3.65± 0.19	5.95± 0.59	6.54± 0.15	12.37± 0.55	13.67± 0.22
Linoleladic(C18:2 Trans)AR g/100g	0.00± 0.00	0.00± 0.00	0.00± 0.00	0.00± 0.00	0.00± 0.00	0.00± 0.00	0.00± 0.00	0.00± 0.00
Linoleic(C18:2 Cis)AR g/100g	0.93± 0.13	1.41± 0.06	5.84± 0.18	5.70± 0.09	4.21± 0.10	4.43± 0.07	1.32± 0.06	1.70± 0.10
Gamma - Linoleic(C18:3 gamma)AR g/100g	0.01± 0.01	0.02± 0.00	0.03± 0.00	0.04± 0.00	0.03± 0.00	0.03± 0.00	0.05± 0.00	0.06± 0.00

Nonadecanoic(C19:0)AR g/100g	0.02± 0.00	0.02± 0.00	0.01± 0.00	0.01± 0.00	0.00± 0.00	0.00± 0.00	0.00± 0.00	0.00± 0.00
Alpha-Linolenic(C18:3 alpha)AR g/100g	0.02± 0.00	0.03± 0.00	0.41± 0.04	0.44± 0.02	0.09± 0.01	0.10± 0.00	0.23± 0.01	0.25± 0.00
Arachidic(C20:0 alpha)AR g/100g	0.01± 0.00	0.00± 0.00	0.03± 0.00	0.03± 0.00	0.02± 0.00	0.02± 0.00	0.03± 0.00	0.04± 0.00
11-Eicosenoic(C20:1)AR g/100g	0.02± 0.01	0.01± 0.00	0.11± 0.01	0.11± 0.01	0.07± 0.01	0.08± 0.00	0.26± 0.11	0.42± 0.01
11-14Eicosenoic(C20:2)AR g/100g	0.03± 0.01	0.04± 0.00	0.11± 0.01	0.11± 0.01	0.07± 0.01	0.07± 0.01	0.06± 0.00	0.06± 0.00
Homo gamama linolenic(C20:3)AR g/100g	0.38± 0.08	0.59± 0.04	0.09± 0.01	0.09± 0.01	0.12± 0.01	0.13± 0.00	0.07± 0.00	0.07± 0.00
11-14-17 Eicosatrienoic(C20:3)AR g/100g	0.01± 0.00	0.02± 0.00	0.11± 0.01	0.11± 0.01	0.00± 0.00	0.01± 0.00	0.00± 0.00	0.00± 0.00
Arachidonic(C20:4)AR g/100g	0.39± 0.06	0.61± 0.01	0.53± 0.03	0.46± 0.03	1.57± 0.22	1.43± 0.06	0.19± 0.01	0.20± 0.00
Heneicosanoic(C21:0)AR g/100g	0.00± 0.00	0.00± 0.00	0.00± 0.00	0.00± 0.00	0.00± 0.00	0.00± 0.00	0.00± 0.00	0.00± 0.00
Behenic(C22:0)AR g/100g	0.00± 0.00	0.00± 0.00	0.00± 0.00	0.00± 0.00	0.00± 0.00	0.00± 0.00	0.00± 0.00	0.01± 0.00
Erucic (C22:1) AR g/100g	0.00± 0.00	0.00± 0.00	0.03± 0.00	0.02± 0.00	0.00± 0.00	0.00± 0.00	0.00± 0.00	0.01± 0.00
Docasadienoic(C22:2)AR g/100g	0.00± 0.00	0.00± 0.00	0.01± 0.00	0.01± 0.00	0.00± 0.00	0.00± 0.00	0.00± 0.00	0.00± 0.00
Docasapentaenoic(C22:5)AR g/100g	0.06± 0.03	0.12± 0.03	0.29± 0.02	0.26± 0.02	0.06± 0.01	0.06± 0.00	0.04± 0.00	0.05± 0.00
Tricosanoic(C23:0)AR g/100g	0.00± 0.00	0.00± 0.00	0.00± 0.00	0.00± 0.00	0.01± 0.00	0.01± 0.00	0.00± 0.00	0.00± 0.00
Lignoceric(C24:0)AR g/100g	0.00± 0.00	0.00± 0.00	0.01± 0.00	0.01± 0.00	0.00± 0.00	0.00± 0.00	0.00± 0.00	0.00± 0.00
Nervonic(C24:1)AR g/100g	0.00± 0.00	0.00± 0.00	0.03± 0.01	0.03± 0.01	0.00± 0.00	0.00± 0.00	0.01± 0.00	0.01± 0.00
Saturated fat (total)AR g/100g	5.09± 0.24	5.81± 0.38	4.00± 0.27	3.93± 0.15	6.74± 0.69	6.96± 0.07	10.59± 0.47	11.67± 0.22
PolySaturated fat (total)AR g/100g	1.91± 0.30	2.93± 0.12	4.07± 0.27	3.72± 0.23	5.28± 0.58	5.36± 0.05	5.49± 0.23	6.01± 0.09
MonoSaturated fat (total)AR g/100g	1.96± 0.35	1.54± 0.15	4.68± 0.37	4.94± 0.27	6.90± 0.68	7.59± 0.17	14.90± 0.67	16.40± 0.25
Omega 9 fatty acids (total)AR g/100g	1.84± 0.32	1.44± 0.14	3.46± 0.24	3.66± 0.19	5.96± 0.59	6.55± 0.14	12.37± 0.55	13.67± 0.22

Nutritional components (Dry weight basis)	FD-DN		FD-PP		F-PP	
	Control	eBeam	Control	eBeam	Control	eBeam
Butyric (C4:0)AR g/100g	0.00± 0.00	0.00± 0.00	0.00± 0.00	0.00± 0.00	0.00± 0.00	0.00± 0.00
Caproic (C6:0)AR g/100g	0.00± 0.00	0.01± 0.00	0.01± 0.00	0.01± 0.01	0.00± 0.00	0.00± 0.00
Caprylic (C8:0)AR g/100g	0.00± 0.00	0.00± 0.00	0.00± 0.00	0.00± 0.00	0.00± 0.00	0.00± 0.00
Capric (C10:0)AR g/100g	0.00± 0.00	0.00± 0.00	0.03± 0.00	0.03± 0.00	0.01± 0.00	0.00± 0.00
Lauric (C12:0)AR g/100g	0.02± 0.00	0.02± 0.00	0.04± 0.00	0.03± 0.00	0.00± 0.00	0.01± 0.00
Tridecanoic(C13:0)AR g/100g	0.00± 0.00	0.00± 0.00	0.00± 0.00	0.00± 0.00	0.00± 0.00	0.00± 0.00
Myristic(C14:0)AR g/100g	0.25± 0.01	0.27± 0.01	0.54± 0.01	0.52± 0.00	0.13± 0.00	0.17± 0.01
Myristoledic(C14:1 Trans)AR g/100g	0.00± 0.00	0.00± 0.00	0.00± 0.00	0.00± 0.00	0.00± 0.00	0.00± 0.00
Myristoleic(C14:1 cis)AR g/100g	0.02± 0.00	0.02± 0.00	0.00± 0.00	0.00± 0.00	0.00± 0.00	0.00± 0.00
Pentadecanoic(C15:0)AR g/100g	0.02± 0.00	0.02± 0.00	0.02± 0.00	0.02± 0.00	0.00± 0.00	0.00± 0.00
Palmitic(C16:0)AR g/100g	5.45± 0.14	5.80± 0.08	9.53± 0.28	9.33± 0.09	2.49± 0.06	3.26± 0.18
Palmiteladic(C16:1 Trans)AR g/100g	0.00± 0.00	0.00± 0.00	0.00± 0.00	0.00± 0.00	0.00± 0.00	0.00± 0.00
Palmitoleic(C16:1 Cis)AR g/100g	0.85± 0.09	0.92± 0.02	1.07± 0.11	1.12± 0.07	2.07± 0.09	2.28± 0.04
Heptadecanoic(C17:0)AR g/100g	0.05± 0.00	0.05± 0.00	0.12± 0.00	0.12± 0.00	0.03± 0.00	0.04± 0.00
10-Heptadecanoic(C17:1)AR g/100g	0.00± 0.00	0.00± 0.00	0.00± 0.00	0.00± 0.00	0.00± 0.00	0.00± 0.00
Stearic(C18:0)AR g/100g	1.62± 0.04	1.72± 0.02	5.63± 0.14	5.51± 0.07	1.36± 0.06	1.74± 0.09
Eliadic(C18:1 Trans)AR g/100g	0.06± 0.00	0.08± 0.00	0.10± 0.00	0.11± 0.00	0.03± 0.00	0.05± 0.00
Oleic(C18:1 Cis)AR g/100g	11.30± 0.29	12.00± 0.15	17.50± 0.58	16.93± 0.13	4.01± 0.05	5.56± 0.30
Linoleladic(C18:2 Trans)AR g/100g	0.00± 0.00	0.00± 0.00	0.00± 0.00	0.00± 0.00	0.00± 0.00	0.00± 0.00
Linoleic(C18:2 Cis)AR g/100g	3.29± 0.34	3.50± 0.02	0.57± 0.06	0.61± 0.04	4.62± 0.20	5.06± 0.08
Gamma - Linoleic(C18:3 gamma)AR g/100g	0.03± 0.00	0.03± 0.00	0.02± 0.00	0.03± 0.00	0.00± 0.00	0.00± 0.00
Nonadecanoic(C19:0)AR g/100g	0.00± 0.00	0.00± 0.00	0.02± 0.00	0.01± 0.00	0.00± 0.00	0.00± 0.00
Alpha-Linolenic(C18:3 alpha)AR g/100g	0.22± 0.01	0.23± 0.00	0.13± 0.06	0.19± 0.01	0.04± 0.00	0.06± 0.01

Arachidic(C20:0 alpha)AR g/100g	0.03± 0.01	0.03± 0.00	0.10± 0.00	0.10± 0.00	0.03± 0.00	0.03± 0.00
11-Eicosenoic(C20:1)AR g/100g	0.28± 0.01	0.30± 0.00	0.42± 0.01	0.42± 0.01	0.10± 0.00	0.13± 0.01
11-14Eicosenoic(C20:2)AR g/100g	0.05± 0.00	0.05± 0.00	0.30± 0.01	0.29± 0.01	0.08± 0.01	0.09± 0.01
Homo gamama linolenic(C20:3)AR g/100g	0.07± 0.00	0.08± 0.00	0.07± 0.00	0.06± 0.00	0.01± 0.00	0.02± 0.00
11-14-17 Eicosatrienoic(C20:3)AR g/100g	0.00± 0.00	0.01± 0.00	0.04± 0.00	0.04± 0.00	0.01± 0.00	0.01± 0.00
Arachidonic(C20:4)AR g/100g	0.52± 0.02	0.51± 0.01	0.69± 0.02	0.70± 0.01	0.15± 0.01	0.14± 0.01
Eicosapentaenoic(C20:5)AR g/100g	0.09± 0.00	0.09± 0.00	0.04± 0.00	0.05± 0.00	0.01± 0.00	0.01± 0.00
Heneicosanoic(C21:0)AR g/100g	0.00± 0.00	0.00± 0.00	0.00± 0.00	0.00± 0.00	0.00± 0.00	0.00± 0.00
Behenic(C22:0)AR g/100g	0.00± 0.00	0.00± 0.00	0.00± 0.00	0.00± 0.00	0.00± 0.00	0.00± 0.00
Erucic (C22:1) AR g/100g	0.00± 0.00	0.00± 0.00	0.00± 0.00	0.00± 0.00	0.00± 0.00	0.00± 0.00
Docasadienoic(C22:2)AR g/100g	0.00± 0.00	0.00± 0.00	0.00± 0.00	0.00± 0.00	0.00± 0.00	0.00± 0.00
Docasapentaenoic(C22:5)AR g/100g	0.05± 0.00	0.05± 0.00	0.06± 0.02	0.09± 0.00	0.02± 0.00	0.02± 0.00
Docasahexaenoic(C22:6)AR g/100g	0.07± 0.00	0.08± 0.00	0.04± 0.00	0.04± 0.00	0.00± 0.00	0.00± 0.00
Tricosanoic(C23:0)AR g/100g	0.02± 0.00	0.02± 0.00	0.00± 0.00	0.00± 0.00	0.00± 0.00	0.00± 0.00
Lignoceric(C24:0)AR g/100g	0.00± 0.00	0.00± 0.00	0.00± 0.00	0.00± 0.00	0.00± 0.00	0.00± 0.00
Nervonic(C24:1)AR g/100g	0.01± 0.00	0.02± 0.00	0.00± 0.00	0.01± 0.00	0.00± 0.00	0.00± 0.00

Vitamins

Nutritional components (Dry weight basis)	FD-BL		FD-WM		FD-CH		FD-GC	
	Untreated	Treated	Untreated	Treated	Untreated	Treated	Untreated	Treated
Beta carotene(Vitamin A)AR IU/Kg	0.00± 0.00	0.00± 0.00	0.00± 0.00	0.00± 0.00	0.00± 0.00	0.00± 0.00	0.00± 0.00	0.00± 0.00
Beta carotene(Vitamin A)DW IU/Kg	0.00± 0.00	0.00± 0.00	0.00± 0.00	0.00± 0.00	0.00± 0.00	0.00± 0.00	0.00± 0.00	0.00± 0.00
Choline chloride AR mg/kg	16733.33± 145.30	16900.00± 288.68	4630.00± 94.52	5213.33± 54.57	6020.00± 47.26	6030.00± 92.92	1850.00± 56.86	1953.33± 6.67
Choline chloride DW mg/kg	18433.33± 133.33	72166.67± 53416.86	4956.67± 96.15	5550.00± 51.32	6310.00± 52.92	6283.33± 90.62	1951.00± 60.86	2046.67± 8.82
Pantothenic acid ARmg/kg	116.67± 6.96	109.57± 8.75	73.33± 2.05	84.07± 5.42	78.87± 1.23	83.80± 3.04	63.80± 1.22	61.40± 2.42
Pantothenic acid DWmg/kg	128.67± 7.88	119.67± 9.82	78.50± 2.17	89.60± 5.76	82.67± 1.33	87.20± 3.15	67.33± 1.11	64.37± 2.48
Pyridoxine (b6) as pyridoxine HCI ARmg/kg	0.58± 0.03	0.55± 0.02	0.01± 0.00	0.01± 0.00	0.10± 0.00	0.10± 0.01	0.05± 0.00	0.06± 0.01
Pyridoxine (b6) as pyridoxine HCI DWmg/kg	0.63± 0.03	0.60± 0.02	0.01± 0.00	0.04± 0.03	0.10± 0.00	0.10± 0.01	0.05± 0.00	0.06± 0.01
Biotin AR mg/kg	0.06± 0.01	0.06± 0.00	0.08± 0.00	0.08± 0.02	0.04± 0.00	0.06± 0.01	0.14± 0.00	0.15± 0.01
Biotin DWmg/kg	0.07± 0.01	0.07± 0.00	0.08± 0.01	0.09± 0.02	0.04± 0.00	0.06± 0.01	0.15± 0.00	0.16± 0.01
Folic acid ARmg/kg	1.59± 0.22	1.84± 0.09	0.03± 0.01	0.06± 0.00	0.05± 0.00	0.09± 0.01	0.05± 0.00	0.07± 0.01
Folic acid DWmg/kg	1.75± 0.25	2.01± 0.10	0.03± 0.01	0.06± 0.00	0.05± 0.00	0.09± 0.01	0.05± 0.00	0.07± 0.01
Vitamin D2 (ergocalciferol) AR IU/kg	0.00± 0.00	0.00± 0.00	37.77± 5.93	0.00± 0.00	0.00± 0.00	0.00± 0.00	0.00± 0.00	0.00± 0.00
Vitamin D2 (ergocalciferol) DW IU/kg	0.00± 0.00	0.00± 0.00	40.40± 6.35	0.00± 0.00	0.00± 0.00	0.00± 0.00	0.00± 0.00	0.00± 0.00
Vitamin D3 (cholecalciferol) AR IU/kg	21.80± 5.80	33.60± 3.01	12666.67± 218.58	11766.67± 578.31	109.67± 12.17	139.00± 15.04	465.33± 9.87	483.67± 12.55
Vitamin D3 (cholecalciferol) DWIU/kg	23.93± 6.32	36.73± 3.27	13566.67± 218.58	12533.33± 600.93	114.83± 12.61	145.00± 15.57	491.33± 9.61	507.00± 13.75
Vitamin E (alpha-tocopherol) AR IU/kg	0.00± 0.00	0.00± 0.00	73.13± 1.29	24.70± 0.62	26.20± 15.13	0.00± 0.00	439.67± 21.18	377.00± 11.93
Vitamin E (alpha-tocopherol) DW IU/kg	0.00± 0.00	0.00± 0.00	78.27± 1.23	26.33± 0.63	27.47± 15.88	0.00± 0.00	464.33± 23.90	395.00± 12.86
Vitamin E (beta-tocopherol) AR IU/kg	0.00± 0.00	0.00± 0.00	0.00± 0.00	0.00± 0.00	0.00± 0.00	0.00± 0.00	0.00± 0.00	0.00± 0.00
Vitamin E (beta-tocopherol) DW IU/kg	0.00± 0.00	0.00± 0.00	0.00± 0.00	0.00± 0.00	0.00± 0.00	0.00± 0.00	0.00± 0.00	0.00± 0.00
Vitamin E (gamma-tocopherol) AR IU/kg	0.00± 0.00	0.00± 0.00	0.00± 0.00	0.00± 0.00	0.00± 0.00	0.00± 0.00	24.83± 0.53	11.37± 5.77
Vitamin E (gamma-tocopherol) DW IU/kg	0.00± 0.00	0.00± 0.00	0.00± 0.00	0.00± 0.00	0.00± 0.00	0.00± 0.00	26.23± 0.55	11.90± 6.05

Vitamin E (delta-tocopherol) AR IU/kg	0.00± 0.00	0.00± 0.00	0.00± 0.00	0.00± 0.00	0.00± 0.00	0.00± 0.00	6.90± 0.23	10.37± 3.77
Vitamin E (delta-tocopherol) DW IU/kg	0.00± 0.00	0.00± 0.00	0.00± 0.00	0.00± 0.00	0.00± 0.00	0.00± 0.00	7.27± 0.23	10.83± 3.94

Nutritional components (Dry weight basis)	FD-DN		FD-PP		F-PP	
	Control	eBeam	Control	eBeam	Control	eBeam
Beta carotene(Vitamin A)AR IU/Kg	366.67± 26.67	310.00± 37.86	563.33± 24.04	506.67± 34.80	0.00± 0.00	0.00± 0.00
Beta carotene(Vitamin A)DW IU/Kg	393.33± 24.04	326.67± 40.55	593.33± 24.04	533.33± 37.56	0.00± 0.00	0.00± 0.00
Choline chloride AR mg/kg	2576.67± 21.86	2766.67± 49.10	4200.00± 75.06	4093.33± 157.20	1610.00± 75.06	1590.00± 15.28
Choline chloride DW mg/kg	2716.67± 21.86	2916.67± 49.10	4440.00± 86.22	4303.33± 172.47	5490.00± 240.21	5503.33± 40.55
Pantothenic acid ARmg/kg	47.77± 0.75	54.93± 0.15	25.27± 0.39	29.23± 2.38	37.67± 0.67	29.37± 1.98
Pantothenic acid DWmg/kg	50.33± 0.81	57.90± 0.15	26.73± 0.47	30.73± 2.58	128.33± 2.60	101.77± 7.03
Pyridoxine (b6) as pyridoxine HCI ARmg/kg	0.15± 0.01	0.15± 0.01	0.32± 0.01	0.33± 0.01	0.21± 0.01	0.17± 0.01
Pyridoxine (b6) as pyridoxine HCI DWmg/kg	0.16± 0.01	0.16± 0.01	0.34± 0.01	0.34± 0.01	0.73± 0.06	0.60± 0.04
Biotin AR mg/kg	0.32± 0.01	0.29± 0.01	0.02± 0.00	0.02± 0.00	0.12± 0.01	0.07± 0.01
Biotin DWmg/kg	0.34± 0.01	0.31± 0.01	0.02± 0.00	0.02± 0.00	0.41± 0.03	0.26± 0.03
Folic acid ARmg/kg	0.07± 0.00	0.30± 0.02	0.11± 0.01	0.59± 0.05	0.35± 0.02	0.71± 0.12
Folic acid DWmg/kg	0.07± 0.00	0.31± 0.02	0.11± 0.02	0.62± 0.05	1.11± 0.08	2.45± 0.43
Vitamin D2 (ergocalciferol) AR IU/kg	0.00± 0.00	0.00± 0.00	0.00± 0.00	0.00± 0.00	0.00± 0.00	0.00± 0.00
Vitamin D2 (ergocalciferol) DW IU/kg	0.00± 0.00	0.00± 0.00	0.00± 0.00	0.00± 0.00	0.00± 0.00	0.00± 0.00
Vitamin D3 (cholecalciferol) AR IU/kg	3646.67± 12.02	3200.00± 36.06	602.67± 12.86	601.67± 18.89	192.33± 7.26	182.00± 4.93
Vitamin D3 (cholecalciferol) DWIU/kg	3843.33± 14.53	3376.67± 39.30	637.33± 11.17	632.67± 19.53	656.67± 30.75	630.00± 21.17
Vitamin E (alpha-tocopherol) AR IU/kg	16.20± 8.18	29.10± 0.75	67.60± 3.80	57.13± 0.97	58.27± 7.92	24.50± 0.93
Vitamin E (alpha-tocopherol) DW IU/kg	17.07± 8.62	30.67± 0.79	71.50± 4.30	60.27± 1.06	197.67± 21.11	84.77± 2.83
Vitamin E (beta-tocopherol) AR IU/kg	0.00± 0.00	0.00± 0.00	0.00± 0.00	0.00± 0.00	0.00± 0.00	0.00± 0.00
Vitamin E (beta-tocopherol) DW IU/kg	0.00± 0.00	0.00± 0.00	0.00± 0.00	0.00± 0.00	0.00± 0.00	0.00± 0.00

Vitamin E (gamma-tocopherol) AR IU/kg	3.13± 0.12	4.23± 0.28	3.47± 0.28	3.20± 0.12	9.00± 0.40	19.87± 3.07
Vitamin E (gamma-tocopherol) DW IU/kg	3.30± 0.15	4.47± 0.32	3.67± 0.28	3.40± 0.12	67.23± 8.32	31.13± 1.28
Vitamin E (delta-tocopherol) AR IU/kg	2.23± 0.07	2.20± 0.06	4.70± 0.10	4.70± 0.15	2.80± 0.26	2.00± 0.06
Vitamin E (delta-tocopherol) DW IU/kg	2.33± 0.07	2.30± 0.06	4.97± 0.12	4.90± 0.15	9.50± 0.64	6.93± 0.26
